# Ammonia production from amino acid-based biomass-like sources by engineered *Escherichia coli*

**DOI:** 10.1186/s13568-017-0385-2

**Published:** 2017-04-20

**Authors:** Yosuke Mikami, Hisanari Yoneda, Yohei Tatsukami, Wataru Aoki, Mitsuyoshi Ueda

**Affiliations:** 1 Division of Applied Life Sciences, Graduate School of Agriculture, Kyoto University, Sakyo-ku, Kyoto, 606-8502 Japan; 2 Japan Society for Promotion of Science, Sakyo-ku, Kyoto, 606-8502 Japan; 3 Kyoto Industrial Science and Technology Innovation Center, Shimogyo-ku, Kyoto, 600-8813 Japan

**Keywords:** Ammonia, Metabolic engineering, *Escherichia coli*, Biorefinery

## Abstract

The demand for ammonia is expected to increase in the future because of its importance in agriculture, industry, and hydrogen transportation. Although the Haber–Bosch process is known as an effective way to produce ammonia, the process is energy-intensive. Thus, an environmentally friendly ammonia production process is desired. In this study, we aimed to produce ammonia from amino acids and amino acid-based biomass-like resources by modifying the metabolism of *Escherichia coli*. By engineering metabolic flux to promote ammonia production using the overexpression of the ketoisovalerate decarboxylase gene (*kivd*), derived from *Lactococcus lactis*, ammonia production from amino acids was 351 mg/L (36.6% yield). Furthermore, we deleted the *glnA* gene, responsible for ammonia assimilation. Using yeast extract as the sole source of carbon and nitrogen, the resultant strain produced 458 mg/L of ammonia (47.8% yield) from an amino acid-based biomass-like material. The ammonia production yields obtained are the highest reported to date. This study suggests that it will be possible to produce ammonia from waste biomass in an environmentally friendly process.

**Electronic supplementary material:**

The online version of this article (doi:10.1186/s13568-017-0385-2) contains supplementary material, which is available to authorized users.

## Introduction

Ammonia is one of the most valuable materials in all aspects of our daily life. Mostly, it is used as a fertilizer in agriculture, and another important usage is as a precursor for nitrogen-containing chemicals such as nitriles, amines, hydrazine, and urea. Additionally, it is anticipated that ammonia has potentiality as a hydrogen liquid carrier in the proposed hydrogen economy (Lan et al. 2012; Miura and Tezuka 2014).

Ammonia synthesis was industrially started by Haber at the beginning of the twentieth century. The Haber–Bosch process now sustains our life by supplying nitrogen fertilizers, keeping up with the increasing demands for food (Erisman et al. 2008). Global production of ammonia reached 146 million tons in 2015 (Gocha 2015). However, we have to consider energy consumption of ammonia production by the Haber–Bosch process because the process needs a lot of energy. The process requires high temperatures (400–600 °C) and high pressures (20–40 MPa), and it is said that more than 1% of the energy generated in the world is used for the Haber–Bosch process (Schrock 2006). Therefore, a more environmentally friendly ammonia production process is desired.

Ammonia production using microorganisms from waste biomass, which is abundant and nitrogen-rich, can be performed at ordinary temperatures and normal pressures. Cleavage of the nitrogen–nitrogen triple bond consumes maximum energy in the Haber–Bosch process. In contrast, nitrogen is already fixed in waste biomass, so ammonia can be easily produced by bacterial dissimilation.

In this study, we show a general concept of ammonia production from waste biomass with metabolically engineered microorganisms. To efficiently produce ammonia, *Escherichia coli* (*E. coli*) was chosen as the host strain because of the accumulated knowledge of its metabolism (Deng et al. 2005; Yim et al. 2011). We planned to direct the amino acid degradation pathway to produce ammonia. First, overexpression of a decarboxylase gene was used as a driving force for ammonia production. Second, we prepared *E. coli* strains that lacked the genes involved in ammonia assimilation, and investigated their influence on ammonia production. By combining these two strategies, we constructed an *E. coli* strain suitable for ammonia production.

## Materials and methods

### Media

Luria–Bertani (LB) medium was prepared with 10 g/L Bacto™ Tryptone (Becton, Dickinson and Company, Detroit, MI, USA), 5 g/L Bacto™ Yeast Extract (Becton) and 10 g/L NaCl (Wako chemicals, Osaka, Japan). M9-YE medium was prepared with 6 g/L K_2_HPO_4_, 3 g/L KH_2_PO_4_, 0.5 g/L NaCl, 7.25 g/L yeast extract, 0.1 mM CaCl_2_, and 1 mM MgSO_4_. In the experiment for the examination of effects of nitrogen sources on ammonia production, Bacto™ Tryptone (Becton), Bacto™ Peptone (Becton), or Bacto™ Casamino acids (Becton) were used instead of Bacto™ Yeast Extract in M9-YE medium. Ampicillin (Meiji Seika Pharma, Tokyo, Japan, 100 μg/mL) and kanamycin (Nacalai Tesque, Kyoto, Japan, 25 μg/mL) were added as appropriate.

### Construction of *E. coli* strains

**Fig. 1:**
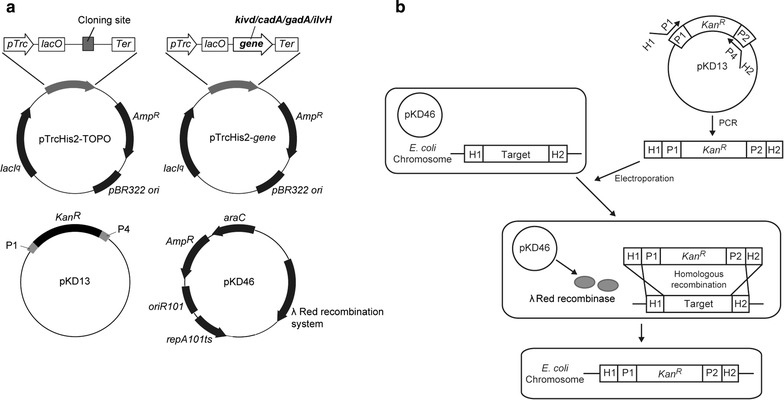
Plasmids for overexpression of decarboxylase and the method for gene deletion in *Escherichia coli*. **a** *Upper side* plasmids for *kivd* overexpression. Control vector (*upper left*) and pTrc-His2-*kivd* (*upper right*). *cadA*, *gadA*, or *ilvH* gene is used instead of *kivd* gene in the right plasmid for the overexpression of these genes. *Lower side* plasmids required for gene deletion in *E. coli*. pKD13 (*lower left*) is a template plasmid for the amplification of kanamycin resistance gene. pKD46 (*lower right*) is a plasmid that increases homologous recombination efficiency in *E. coli* chromosome. **b** A simple gene knockout strategy by homologous recombination. *P1 or P2* priming site, *H1 or H2* homologous recombination site

All primers and strains used in this study are listed in Table 1 and Additional file 1: Table S1, respectively. To clone the *kivd* gene, which encodes keto acid decarboxylase, genomic DNA of a *Lactococcus lactis* subsp. (ATCC 19435D-5, purchased from the ATCC), was used as a PCR template, with primers YN31/YN32. To clone *cadA*, *gadA*, or *ilvH* genes, firstly genomic DNA of *E.coli* DH5α (Thermo Fisher Scientific) was extracted using the Genomic-tip 100/G kit (QIAGEN, Hilden, Germany). Next, the genomic DNA was used as a template for PCR amplification with primers YN33/YN34, YN35/YN36, or YN39/YN40, respectively. Resultant PCR products were cloned into the pTrcHis2-TOPO^®^ vector (Thermo Fisher Scientific, Waltham, MA, USA) by TA-cloning (Fig. 1a). The control strain was *E. coli* DH10B; harboring the plasmid pTrcHis2-TOPO^®^/*lacZ* (Thermo Fisher Scientific). Protein production was validated by sodium dodecyl sulfate–polyacrylamide gel electrophoresis (SDS-PAGE) using a 5–20% e-PAGEL (ATTO, Tokyo, Japan) and Full Range RPN800E protein markers (GE healthcare, Little Chalfont, UK). Proteins on the gel were stained with Coomassie Brilliant Blue (Bio-Rad, Hercules, CA, USA).

**Table 1:** Strains and plasmids

Strains or plasmids	Genotype or description	Reference
*E. coli* strains		
DH10B	F–*mcr*A Δ(*mrr*-*hsd*RMS-*mcr*BC) Ф80*lac*ZΔM15Δ*lac*X74*rec*A1*end*A1*ara*D139 Δ(*ara leu*) 7697*gal*U*gal*K*rps*L*nup*G λ–	Thermo Fisher Scientific
DH10B*ΔglnA*	DH10B *ΔglnA:*: FRT-*kan* ^*R*^-FRT	This study
DH10B*ΔgdhA*	DH10B *ΔgdhA:*: FRT-*kan* ^*R*^-FRT	This study
Plasmids		
pTrcHis2-TOPO	P_trc_:: TOPO site, pBR322 ori*, lacI* ^*q*^, *Amp* ^*R*^	Thermo Fisher Scientific
Control plasmid (pTrcHis2-*lacZ*)	P_trc_:: *lacZ*, pBR322 ori, *lacI* ^*q*^, *Amp* ^*R*^	Thermo Fisher Scientific
pTrcHis2-*kivd*	P_trc_:: *kivd*, pBR322 ori, *lacI* ^*q*^, *Amp* ^*R*^	This study, Accession number: AJ746364.1
pTrcHis2-*cadA*	P_trc_:: *cadA*, pBR322 ori, *lacI* ^*q*^, *Amp* ^*R*^	This study, Accession number: AY319765.1
pTrcHis2-*gadA*	P_trc_:: *gadA*, pBR322 ori, *lacI* ^*q*^, *Amp* ^*R*^	This study, Accession number: EF547388.1
pTrcHis2-*ilvH*	P_trc_:: *ilvH*, pBR322 ori, *lacI* ^*q*^, *Amp* ^*R*^	This study, Accession number: EG10499
pKD46	Red recombinase expression plasmid	Datsenko et al. (2000)
pKD13	*Kan* ^*R*^ template plasmid	Datsenko et al. (2000)

Gene deletion was performed using a homologous recombination system using lambda Red proteins according to the method by Datsenko (Fig. 1b) (Datsenko and Wanner 2000). The plasmid and method for gene deletion are shown in Fig. 1a and b, respectively.

### Ammonia production

All cultivations were performed in a shaker (TAITEC, Saitama, Japan) at 165 rpm and 37 °C. For pre-culture, *E. coli* strains were cultivated in 5 mL LB medium at 37 °C overnight. The overnight culture was inoculated into 2.5 mL of M9-YE medium, or other media, to achieve an initial OD_600_ value of 0.5. Cells were grown for 2.5 h before adding 0.1 mM (final concentration) of isopropyl-β-d-thiogalactoside (IPTG). After adding IPTG, cells were grown for 24 h. Ammonia meter (Ion meter TiN-9001, Toko Chemical Laboratories, Tokyo, Japan) was used to measure ammonia dissolved in the culture supernatant. Ammonia yields in M9-YE medium and other nitrogen-containing media were calculated based on the nitrogen contents in the BD Bionutrients™ Technical Manual Third edition (BD Biosciences 2015). Referring to the manual, for example, the theoretical maximum ammonia production in M9-YE medium (containing 7.25 g/L of yeast extract) was calculated to be 960 mg/L.

## Results

### Overexpression of 2-keto acid decarboxylase genes

**Fig. 2:**
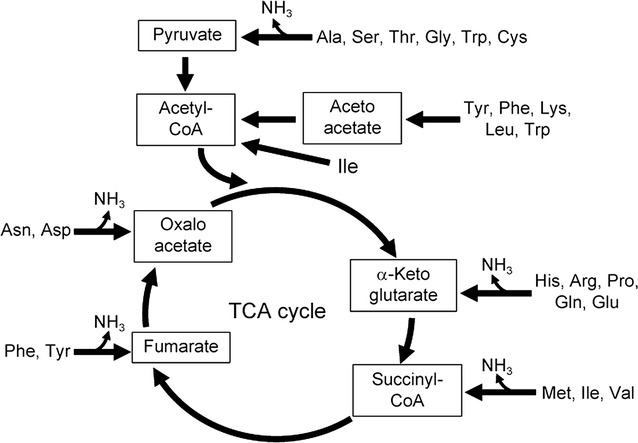
Catabolic pathway of amino acids

**Fig. 3:**
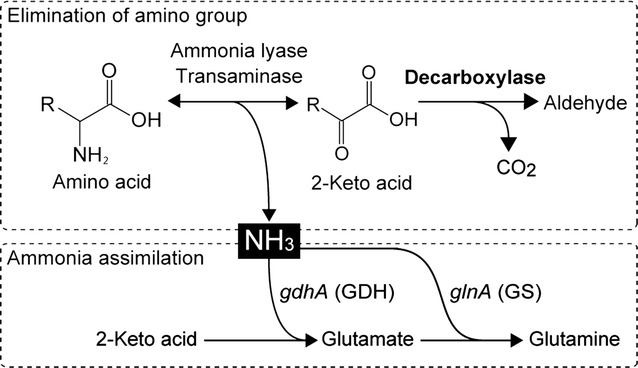
Scheme of ammonia production. Elimination process of amino groups (*upper*). Assimilation process of amino groups (*lower*)

To produce ammonia from amino acids, we planned to modify catabolism of amino acids in *E. coli*. The catabolic pathway of amino acids in *E. coli* is shown in Fig. 2. Natively, ammonia lyases such as *ilvA* (Eisenstein 1990), or transaminases such as *ilvE* (Inoue et al. 1988) catalyze the elimination of amino groups from amino acids. As a result of the elimination of amino groups, 2-keto acids are produced. Thus, we hypothesized that an irreversible decarboxylation of these 2-keto acids could be a driving force to engineer the metabolic flux toward ammonia production (Fig. 3, upper).

First, we examined the effect of selected decarboxylases on ammonia production; *kivd* (α-ketoisovalerate decarboxylase) (de la Plaza et al. 2004), *gadA* (glutamate decarboxylase) (Waterman and Small 2003), *cadA* (lysine decarboxylase) (Lemonnier and Lane 1998), and *ilvH* (acetolactate synthase) (Defelice et al. 1974). In these experiments, ammonia production was performed in M9-YE medium using yeast extract as the sole source of carbon and nitrogen. We used yeast extract as a model of waste biomass because it contains abundant amino acids as a nitrogen source (Biosciences 2015). As a result, we found that the *kivd*-overexpressing *E. coli* strain achieved the highest ammonia production (351 mg/L, 36.6% yield), while the control *E. coli* strain produced some ammonia using the native ammonia metabolism (Table 2).

**Table 2:** Growth and ammonia production of decarboxylase-overexpressing *E. coli* strains in M9_YE medium

Sample	Cell density OD_600_	Produced ammonia by *E. coli* (mg/L)	Theoretical amount of maximum ammonia production (mg/L)^a^	Yield (%)
Medium only	0.0253 ± 0.0073	24.2 ± 2.7	960	2.50
Control strain	3.73 ± 0.05	260 ± 11	960	27.1
*kivd*	2.31 ± 0.09	351 ± 5	960	36.6
*cadA*	5.11 ± 0.03	230 ± 6	960	24.0
*gadA*	2.89 ± 0.01	256 ± 6	960	26.7
*ilvH*	5.61 ± 0.07	239 ± 26	960	24.9

Values of yield were calculated as produced ammonia by *E. coli* per theoretical amount of maximum ammonia production. Values given as mean ± SD (n = 3)

^a^The values were calculated based on the nitrogen contents of BD Bionutrients™ Technical Manual Third edition (http://www.bd.com/ds/technicalCenter/misc/lcn01558-bionutrients-manual.pdf.)

### Gene deletion of *E. coli* to improve ammonia production

To improve the efficacy of ammonia production, we deleted genes involved in ammonia assimilation. We chose two genes, *glnA* (glutamine synthetase) and *gdhA* (glutamate dehydrogenase), as knockout candidates to produce increased amounts of ammonia (Fig. 3, lower). Glutamate and glutamine are the major products of ammonia assimilation and they serve as intracellular nitrogen donors. Glutamine synthetase (GS) and glutamate dehydrogenase (GDH) assimilate most of the ammonia in *E. coli*, according to following reactions (van Heeswijk et al. 2013):

$${\text{GDH }}\left( {gdhA} \right):\upalpha{\text{-Ketoglutarate}} + {\text{NH}}_{ 3} + {\text{NADPH}} \to {\text{Glutamate}} + {\text{NADP}}^{ + }$$

$${\text{GS }}\left( {glnA} \right):{\text{ Glutamate}} + {\text{NH}}_{ 3} + {\text{ATP}} \to {\text{Glutamine}} + {\text{ADP}} + {\text{Pi}}$$

**Fig. 4:**
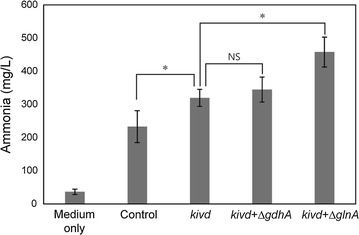
Ammonia production of modified strains. Values given as mean ± SD (n ≥ 3). *Statistical significances are determined by Tukey test (P < 0.01). *NS* no significance

Deletion of *glnA* (Δ*glnA*) or *gdhA* blocks ammonia re-uptake and lead to secretion of ammonia into the media. Therefore, we individually deleted each of these genes in *E. coli* to try to improve ammonia yield. The amount of ammonia produced by these gene-deleted strains was evaluated in combination with the overexpression of *kivd* in M9-YE medium. As shown in Fig. 4, ammonia production reached 458 mg/L (47.8% yield) when the overexpression of *kivd* and deletion of *glnA* was combined. The reason why ammonia production did not improve in the *gdhA* deleted strain (Fig. 4) was thought to be a property of GDH. GDH has low affinity for ammonia (K_M_ = 1–2 mM) (Sharkey and Engel 2008), while GS has high affinity (K_M_ = 0.1 mM) (van Heeswijk et al. 2013). Hence, GDH is less efficient in low ammonia concentrations, such as in M9-YE medium.

Next, we examined the effect of nitrogen source on ammonia production. Efficiency of ammonia production is affected by nitrogen content and difficulty of decomposition. Four media were used for ammonia production by the *kivd* + Δ*glnA* strain (Table 3). The yield in M9-YE medium was higher than that in the other media, except for the medium using casamino acids. The yields from peptone or tryptone media were low, probably because they contain long-chain peptides that *E. coli* cannot degrade (Huo et al. 2011). The ammonia yield from casamino acids was slightly higher than that from yeast extract (Table 3). The reason may be that the content of amino nitrogen per total nitrogen in casamino acids is higher than that in yeast extract (Table 4), leading to efficient ammonia production in casamino acid-based medium by *E. coli*.

**Table 3:** Ammonia production by the *kivd* + Δ*glnA* strain with various nitrogen sources (7.25 g/L)

Nitrogen source	Cell density OD_600_	Produced ammonia by *E. coli* (mg/L)	Theoretical amount of maximum ammonia production (mg/L)^a^	Yield (%)
Yeast extract (M9_YE)	1.51 ± 0.05	439 ± 12	960	45.8
Tryptone	1.25 ± 0.08	427 ± 25	1171	36.5
Peptone	1.00 ± 0.06	375 ± 10	1356	27.7
Casamino acids	0.99 ± 0.03	453 ± 35	951	47.6

Values of yield were calculated as produced ammonia by *E. coli* per theoretical amount of maximum ammonia production. Values given as mean ± SD (n = 3)

^a^The values were calculated based on the nitrogen contents of BD Bionutrients™ Technical Manual Third edition (http://www.bd.com/ds/technicalCenter/misc/lcn01558-bionutrients-manual.pdf.)

**Table 4:** Compositions of various nitrogen-containing sources

Nitrogen source	Total nitrogen [TN, % (w/w)]	Amino nitrogen [AN, % (w/w)]	AN/TN (%)	Total amino acids [% (w/w)]	Free amino acids [% (w/w)]	Free amino acids/total amino acids (%)
Yeast extract	10.9	6.0	55.0	52.2	32.8	62.8
Tryptone	13.3	5.3	39.8	76.4	26.9	35.2
Peptone	15.4	3.5	22.7	72.4	14.8	20.4
Casamino acids	10.8	9.4	87.0	62.8	51.0	81.2

The values were calculated based on the nitrogen contents of BD Bionutrients™ Technical Manual Third edition (http://www.bd.com/ds/technicalCenter/misc/lcn01558-bionutrients-manual.pdf). Total amino acids include forms of protein and peptide

## Discussion

The demand for ammonia is expected to grow in the future. Recent studies examined ammonia production using microorganisms such as nitrogen-fixing bacteria (Higo et al. 2016). In this study, we focused on waste biomass, which has abundant nitrogen-containing compounds. By engineering ammonia metabolism in *E. coli*, the best strain produced 458 mg/L of ammonia from 7.25 g/L of yeast extract (Fig. 4). This ammonia yield is 23% higher than that in the previous study (Choi et al. 2014). Our study demonstrated that ammonia could be recovered in a low energy-consuming manner and that it would be possible to cover some of the increasing demand for ammonia in the future.

As shown in Table 4, yeast extract (Becton) was selected as the nitrogen source because more than 60% of nitrogen-containing compounds in yeast extract are easily degradable amino acids. The yield of ammonia is affected by the form of nitrogen in a medium. As shown in Table 3, the ammonia yield in M9-YE medium was higher than the media using peptone or tryptone, and equivalent to the medium using casamino acids. While yeast extract or casamino acids contain a lot of amino acids, peptone and tryptone consist of proteins and long-chain peptides (Table 4). Because *E. coli* cannot directly utilize proteins and long-chain peptides as a nutrient source, they must be decomposed into short-chain peptides before ammonia production (Huo et al. 2011). This is done by heterologous expression of strong proteases in *E. coli* (Choi et al. 2014; Su et al. 2012).

The ammonia yield with the best strain (*kivd* + Δ*glnA*) reached 47.8%. The remaining 52.2% was thought to be used for cell biomass or not catabolized. In order to increase the yield of ammonia, it is necessary to balance the growth of *E. coli* and ammonia production. The more *E. coli* grows, the less ammonia is produced in media, because overgrowth of *E. coli* leads to accumulation of ammonia in the form of cell biomass. Optimization of the balance between growth and ammonia production will be required to achieve the best yield. There have been studies trying to produce chemicals efficiently with the minimum growth required for production (Soma et al. 2014; Brockman and Prather 2015). For example, Soma et al. constructed two modes, a growth mode and a production mode, and engineered cells transitioned from growth mode to production mode automatically in culture. Strategies taken in these studies can be applied to increase ammonia yield.

## Additional file

**Additional file 1: Table S1.** Primers.

## Abbreviations

*E. coli*: *Escherichia coli*
SDS-PAGE: sodium dodecyl sulfate–polyacrylamide gel electrophoresis
IPTG: isopropyl-β-d-thiogalactoside
GS: glutamine synthetase
GDH: glutamate dehydrogenase
*B. subtilis*: *Bacillus subtilis*
