# Supplementary material for: Ammonia production from amino acid-based biomass-like sources by engineered Escherichia coli
Source: AMB Express. 2017 Apr 20;7:83. doi: 10.1186/s13568-017-0385-2 (PMC5399010; doi:10.1186/s13568-017-0385-2)
Supplement: Supplementary file 1 — Additional file 1: Table S1. Primers. [file 13568_2017_385_MOESM1_ESM.docx]

Table S1 Primers

| Primer | Sequence 5’-3’ | Description |
| --- | --- | --- |
| YN31 | TATACAGTAGGAGATTACCTATTAG | Forward primer for *kivd* cloning |
| YN32 | TGATTTATTTTGTTCAGCAAATAG | Reverse primer for *kivd* cloning |
| YN33 | AACGTTATTGCAATATTGAATC | Forward primer for *cadA* cloning |
| YN34 | TTTTTTGCTTTCTTCTTTCAATACC | Reverse primer for *cadA* cloning |
| YN35 | GACCAGAAGCTGTTAACGGATT | Forward primer for *gadA* cloning |
| YN36 | GGTGTGTTTAAAGCTGTTCTG | Reverse primer for *gadA* cloning |
| YN39 | GAGATGTTGTCTGGAGCCGAG | Forward primer for *ilvI-H* cloning |
| YN40 | ACGCATTATTTTATCGCCGCGCGAA | Reverse primer for *ilvI-H* cloning |
| YN53 | GCCAGAGACAGGCGAAAAGTTTCCACGGCAACTAAAACACGTGTAGGCTGGAGCTGCTTC | Forward primer for *glnA* disruption |
| YN54 | GTTACCACGACGACCATGACCAATCCAGGAGAGTTAAAGTCTGTCAAACATGAGAATTAA | Reverse primer for *glnA* disruption |
| YN55 | CATAAGCACAATCGTATTAATATATAAGGGTTTTATATCTGTGTAGGCTGGAGCTGCTTC | Forward primer for *gdhA* disruption |
| YN56 | TGTAGGCCTGATAAGCGTAGCGCCATCAGGCATTTACAACCTGTCAAACATGAGAATTAA | Reverse primer for *gdhA* disruption |
